# Use of Medicinal Plants in the Process of Wound Healing: A Literature Review

**DOI:** 10.3390/ph17030303

**Published:** 2024-02-27

**Authors:** Mayra Cedillo-Cortezano, Luis Ruben Martinez-Cuevas, Jesús A. Márquez López, Ingrid L. Barrera López, Samantha Escutia-Perez, Vera L. Petricevich

**Affiliations:** Facultad de Medicina, Universidad Autónoma del Estado de Morelos (UAEM), Cuernavaca 62350, Mexico; mayra.cedillo@uaem.edu.mx (M.C.-C.); luis.martinez@uaem.edu.mx (L.R.M.-C.); jesus.marquezlop@uaem.edu.mx (J.A.M.L.); ingrid.barreralop@uaem.edu.mx (I.L.B.L.); samantha.escutiaper@uaem.edu.mx (S.E.-P.)

**Keywords:** plant extracts, phytotherapy, treatments, healing, medicinal plants, secondary metabolites

## Abstract

The literature on the use of medicinal plants in wound healing was comprehensively searched to obtain and assess the data. The data were procured via clinical studies that utilized medicinal plants and their compounds in vitro and in vivo for wound healing. This review collected data from electronic databases, including Google Scholar, PubMed, Science Direct, Web of Science, SciFinder, Thesis, and Scopus, using the search terms “natural products”, “wound healing”, and “natural compounds”, along with the keywords “plants”, “extracts”, and “phytochemicals”. Results from the last decade reveal a total of 62 families and 109 genera of medicinal plants, and their compounds have been studied experimentally both in vivo and in vitro and clinically found to effectively promote healing. This activity is related to the presence of secondary metabolites such as flavonoids, alkaloids, saponins, tannins, terpenoids, and phenolic compounds, which act at different stages through different mechanisms to exert anti-inflammatory, antimicrobial, and antioxidant effects, confirming that the use of medicinal plants could be an adequate alternative to current conventional practices for treating wounds.

## 1. Introduction

Statistics derived by the World Health Organization (WHO) have shown that around 80% of the world’s population uses traditional medicine for primary healthcare, and 85% of this group utilizes plants. One of the great challenges of modern medicine concerns the healing and treatment of wounds. Studies by the WHO show that around 5 million people die annually because of imperfect wound healing. The use of natural products has shown promise in preventing and treating wounds. This review aims to elucidate the modes of preparation of herbal treatments, their phytochemical contents, and their use in formulations for wounds. Plants show a broad spectrum of bioactive phytochemicals, categorizable into the families of alkaloids, carotenoids, phenolic compounds, steroids, flavonoids, saponins, tannins, and terpenoids. These compounds act at different phases of the healing process through different mechanisms and show anti-inflammatory, antimicrobial, and antioxidant effects, whilst promoting collagen synthesis, cell proliferation, and angiogenesis. The application of natural compounds via new systems can contribute to enhancements in wound treatment.

## 2. Methods

### 2.1. Information Sources, Searching, and Selection of Studies 

For this review, the period considered was January 2013 to October 2023, and the electronic databases used include Google Scholar, PubMed, Scopus, Science Direct, Web of Science, SciFinder, and Theses. The terms used for the search were “wound healing” (sought in the titles and abstracts) and the keywords “plant”, “extract”, “natural compounds”, and “phytochemicals”. We also manually searched for references to bioactive phytochemicals that act at different phases of the healing process through different mechanisms and have anti-inflammatory, antimicrobial, antioxidant, and cell proliferation-promoting effects. The full documents were read to verify that they met the inclusion criteria.

### 2.2. Eligibility Criteria

Inclusion criteria: Studies published in English, including theses, articles, and proceedings from January 2013 to 01 October 2023, with “wound healing” in the title or abstract, addressing experimental or clinical studies. 

Exclusion criteria: Newspapers and reviews.

### 2.3. Results

Following the initial screening, we identified approximately 5000 articles in different databases, citing about 480 different genera of plants with healing activities. Only 22% of these were included in this study, in accordance with the eligibility criteria. This is the first study in the last ten years to address plants and major and/or new compounds with regard to their activity in wound healing. The results obtained from eligible studies reveal a total of 62 families and 109 genera of medicinal plants used for wound treatment that have been discussed in studies from the last 10 years. Their effects, attributed to flavonoids, alkaloids, saponins, and phenolic compounds, which act at different stages through different mechanisms, include anti-inflammatory, antimicrobial, and antioxidant effects. 

## 3. Classification of Wounds

The Healing Society defines wounds as physical lesions resulting from an opening or breaking of the skin that causes disturbances within the anatomy and normal functioning of the skin [1,2]. Wound healing can be a complex process because it entails a series of interdependent and overlapping stages: inflammation (exudative phase), reconstruction (proliferative phase), epithelization (regenerative phase), and maturation [3].

Several factors may affect the healing process, including (a) the presence of a contaminated surface contacting the wound; (b) delays due to the consumption of infected nutrients as a source of energy by white blood cells; (c) associated illnesses, such as diabetes and morbid obesity, which cause hyperglycemia and thus impact the defense mechanisms of the body, impairing the capacities of white blood cells in general, and especially neutrophils; and (d) treatment with radio-chemotherapy, NSAIDs and immunosuppressive drugs [4,5,6]. Wounds can be classified in various ways based on their etiology, their position, the kind of injury, the associated changes in bodily function, the wound depth, tissue loss, or clinical appearance. Table 1 describes the classification of a wound.

### 3.1. Healing Process

Wounds can change the physiology of the skin, particularly those that affect the dermal layer. Therefore, tissue lesions can modify the anatomical structure of the skin, and the degree of damage to the tissue is highly dependent upon the healing mechanism. The wound-healing process entails a cascade of cellular and molecular events aimed at restoring the injured area [7].

**Figure 1:**
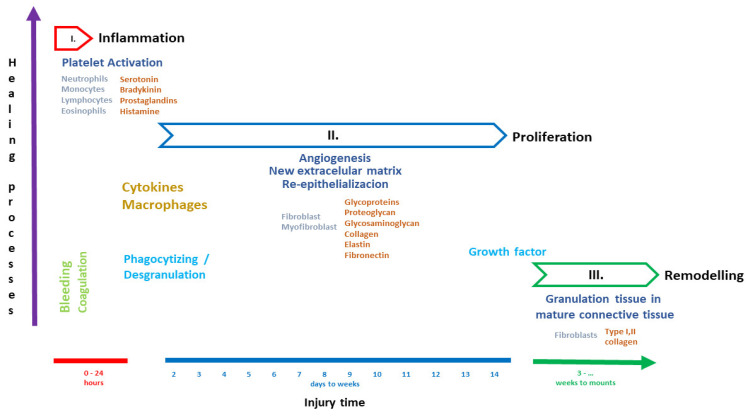
Wound-healing stages.

The healing cascade is an organized sequence of events, and classifications have been applied to it to facilitate our understanding of the dynamic processes it involves that closely determine healing [8,9]. Different authors divide up the healing process in different ways; some consider the initial step to involve inflammation, followed by proliferation and ending with repair in the remodeling stage [7,8,9] (Figure 1).

### 3.2. Inflammation

Inflammation is a defensive reaction to harmful agents, including microorganisms and damaged cells produced by the body, and promotes biological processes such as vascular responses and systemic reactions intended to reestablish the equilibrium of tissue homeostasis. In the absence of the inflammatory process, infections would develop in an uncontrolled manner; thus, the destructive processes unfolding in organs would continue until a total loss of function. The inflammatory process can be assessed clinically using five classical signs, called the “Cardinal Signals”: swelling, heat, redness, pain, and loss of function [10,11]. 

The major function of the inflammatory response is the conduction of leukocytes to the affected region, which play an important role in defense by phagocytizing or producing substances that destroy microorganisms and necrotic tissues; they can inactivate or degrade antigens. Circulating cells such as neutrophils, monocytes, lymphocytes, and eosinophils reach the site of aggression through the bloodstream, crossing the vessel wall and migrating toward the site of aggression in significant quantities during the first 24 h of injury. The circulating cells are attracted by the notable inflammatory cytokine effects produced by activated platelets, endothelial cells, and the degraded products of the pathogens present in the lesion [12,13].

These chemical mediators can cause the dilation of the arterioles and increase the permeability of capillaries and venules, allowing a greater flow of blood to the damaged area, as well as the exudation (extravasation) of liquids, proteins, and defense cells into the interstitial space. Fluid exudation results in inflammatory edema, whereby the blood gradually becomes more viscous due to the increased density of red blood cells, and the circulation of the small vessels gradually slows, ultimately culminating in blood stasis (small, dilated vessels filled with red blood cells). At the same time, leukocytes migrate through the vascular wall into the interstitial space (called transmigration, diapedesis, or leukocyte emigration); this constitutes the initial (acute) phase of any type of inflammatory response, also referred to as the vascular phenomena [12,14,15]. 

The recruitment and activation of inflammatory cells, in either acute or chronic inflammation, are consequences of physical changes occurring at the wound site. The first of these changes is the liberation of substances following platelet degranulation. These include thrombin, which prompts the release of distinct growth factors such as platelet-derived growth factors (PDGFs), transforming growth factor-β (TGF-β), epidermal growth factor (EGF), transforming growth factor-α (TGF-α), and endothelial cell growth factor (VEGF). The mediators mentioned beforehand include adhesive glycoproteins such as fibronectin and thrombospondin—important constituents of the extracellular matrix [16].

The inflammatory phase of wound healing includes the activation of vasoactive substances such as serotonin, bradykinin, prostaglandins, and histamine. These can increase the permeability of the endothelium at the lesion site and enhance interstitial fluid perfusion in this area. The increase in permeability facilitates infiltration by immune and repair cells that facilitate the previously described events, while the increase in circulation leads to greater oxygen distribution in the tissue; consequently, the temperature increases at the site of injury. The warm and humid microenvironment thus produced within the wound is essential for the ensuing healing phase. At the end of the inflammatory stage of wound healing, macrophages synthesize distinct growth factors, such as PDGF, TGF-β, fibroblast growth factor (FGF), and VEGF, which stand out as the major cytokines required to stimulate the formation of granulation tissue and thus generate the environment required for the next phases of cell proliferation and repair [1,17]. 

### 3.3. Proliferation 

The closure of the lesion occurs in the proliferation phase. Angiogenesis occurs as a result of the formation of granulation tissue and is responsible for filling the injured tissue. The new extracellular matrix that will be involved in cell growth and the new blood vessels that will convey oxygen and nutrients indispensable for local cellular metabolism are produced with the aid of fibroblasts. With the progression of the proliferative phase, the provisional matrix changes as a result of the newly formed granulation tissue. Wound epithelialization represents the final stage of the proliferative phase [18,19].

The formation of the extracellular matrix provides a substrate for cell adhesion and regulates the growth, movement, and differentiation of cells within it. The extracellular matrix consists of structural proteins, including collagen and elastin, along with an interstitial matrix composed of adhesive glycoproteins, proteoglycan, and glycosaminoglycan [20,21].

The increase in microvascular permeability, characteristic of the inflammatory process, represents the first stage of this proliferative process; here, cellular elements, along with cytokines, are released, and we also see the formation of the provisional extracellular matrix necessary for the migration and proliferation of endothelial cells [22]. 

Angiogenesis is an exceedingly important stage in the healing process, during which new blood vessels are formed from preexisting vessels. The new vessels participate in the formation of provisional granulation and the supply of nutrients and oxygen to the growing tissue. On the other hand, vasculogenesis refers to the early stages of vascular development, during which vascular endothelial precursor cells enact the mobilization of endothelial progenitors derived from bone marrow [23]. 

Fibroblast migration is induced by the PDGF and TGF-β released by macrophages. When fibroblasts reach the wound bed, they proliferate and produce matrix proteins such as fibronectin, collagen, and proteoglycans. These components help build the new extracellular matrix, which supports the further growth of cells essential for the repair process. A crucial interaction takes place between the fibroblasts and extracellular matrix, which regulates the additional synthesis of the components along with tissue remodeling [24]. 

The process of re-epithelialization in the injured tissue is accelerated by the contraction of the underlying connective tissue, which is responsible for the approximation of the wound’s margins. This contraction is induced by myofibroblasts, activated by TGF-β and PDGF; thus, these myofibroblasts play an important role in wound healing, especially for open lesions. When present in open wounds, these cells produce larger amounts of extracellular matrix components. However, if abnormalities arise in the physiological process, such as delays, this may cause cicatricial defects due to alterations to the differentiation of fibroblasts in myofibroblasts [25].

### 3.4. Remodeling

The final stage of wound healing involves the remodeling or maturation of granulation tissue into mature connective tissue or scar tissue. The wound-healing process is most potent during this phase. Wound maturation begins during the third week after the wounding and is characterized by an increase in resistance and a controlled decrease in the amount of collagen. This mechanism is characterized by a balance in the production and destruction of collagen fibers, resulting from the action of an enzyme called collagenase. An imbalance in this relationship can favor the emergence of hypertrophic and keloid scars [1,2]. 

Finally, the remodeling process consists of the proper deposit of elements previously mentioned, mainly including collagen fibers. This stage involves a change in the type of collagen present and its disposition. Type III collagen, which is initially more abundant in the wound than type I, is more actively degraded over time; in contrast, the production of collagen I by fibroblasts increases, and this causes an increase in tension force and the reduction in the quantity of collagen [3,4].

Numerous factors can aggravate the wound-healing process, specifically as regards the biological events that comprise it. Factors such as advanced age, the patient’s nutritional status, and vascular changes can directly alter the healing process. However, diabetes mellitus also drastically alters the process of tissue recovery, interfering at all stages and thus causing serious complications for the patient. 

The process of wound healing is complicated when the patient is diabetic. In patients with diabetes, wounds show less revascularization and lower expression of growth factors compared to injuries in non-diabetics, thus impairing healing. These complications can evolve to produce severe consequences, such as a stagnant repair mechanism leading to a loss of tissue function [26,27,28].

Impaired healing in diabetic patients is characterized by acute inflammation and abnormalities in angiogenesis, entailing difficulties in forming new blood. The proper healing of a wound requires a regulated inflammatory response; however, diabetic wounds show prolonged inflammatory responses. Wounds in DM1 exhibit increased expression of inflammatory cytokines, including tumor necrosis factor alpha (TNF-α) and interleukins IL-1 and IL-6, and decreased IL-10, leading to injury following a prolonged inflammatory phase. This deregulated and prolonged inflammation leads to the wound becoming chronic and unable to be completely healed [29]. These chronic injuries, such as foot ulcers (diabetic foot), lead to high morbidity and increased treatment costs. In addition, foot ulcers substantiate more than 50% of the cases of amputation among diabetics. Increased oxidative stress is one of the leading causes of wound complications in diabetics, causing late scarring. Reducing persistent inflammation and the excretion of free radicals by incorporating an anti-inflammatory and antioxidant agent into wound treatment has become an important strategy for improving the healing of diabetic wounds [30]. 

Factors associated with both angiogenesis and the vasculogenesis process are vital for wound healing, as they play a vital role in the delivery of oxygen, nutrients, and other mediators to the wound site. Thus, they have become therapeutic targets that can improve the healing of damaged wounds in diabetes patients when activated, thus restoring the neovasculogenesis mechanism [31].

## 4. Medicinal Plants Used for Wound Healing

Preparations made using medical plants (such as extracts) and the active compounds present in some of these plants have been used to accelerate wound healing. The ethnopharmacological approach to investigating medicinal plants consists of combining information acquired from users of medicinal flora (traditional communities) with the results of chemical and pharmacological studies [32]. 

The application of medicinal plants has always been a part of the evolution of humanity; these plants represent one of the first therapeutic resources to be used by humans, and they still hold great importance for the maintenance of human health. According to the World Health Organization (WHO, 2002) [33], approximately 80% of the population in developing countries use traditional medicine as their primary healthcare, most of which entails using plant extracts or their active compounds. According to the statistics provided by the WHO, medicinal plants, herbal preparations, or derived products are conventionally used in primary care in various countries. The WHO classifies a medicinal plant as a plant species that, when administered to humans, exerts a pharmacological action. The findings of ethnopharmacology, in terms of the therapeutic properties of plants and popular knowledge regarding their usage, have been presented as source material for developing technical scientific knowledge. The accumulation of information regarding the use of natural assets by traditional populations has provided researchers with models for the sustainable use of these resources while also providing directions for the exploitation of the pharmacological properties of certain species. Over the centuries, products of plant origin have been commonly used as the basis of treatments for different diseases by virtue of knowledge transmitted down through generations, and certain plant species can be understood as sources of active molecules [34,35,36]. 

In the context of wound healing, the utilization of plants and plant extracts dates to the prehistoric era [37]. Records describe the use of plants and extracts in the form of poultices to stop hemorrhages and to facilitate cicatrization. Other uses have been described in relation to the ingestion of certain plants, which act systemically [36]. Thus, the data collected through the years confirm that the development of modern medicine has only been possible via the inheritance of ancient healing methods and the empirical knowledge pertaining to such practices [37]. Every year, approximately 100 million patients around the world acquire scars resulting from surgical interventions, burns, or tissue ruptures due to accidents of various kinds, which require effective and rapid treatment. These statistics indicate that wound healing is a modern therapeutic challenge [38]. Multiple studies have sought to improve the treatment of wounds by promoting the healing process; nevertheless, the most effective organic and inorganic substances in this regard remain a scientific mystery to this day [39]. Healing involves several complex processes in which different cellular structures are involved. The process begins with an amplified immune response that prevents wound complications, enacted via chemoattraction, which facilitates the development of other mediators necessary to subsequent phases, such as inflammation, cell proliferation, and re-epithelization, which eventually lead to wound closure [40].

Medicinal plants are significant sources of novel chemical substances with valuable therapeutic effects. Table 2 displays the families and genera of plants utilized for wound healing. A total of 62 families and 109 genera were documented with applicability in wound healing and treatment based on traditional medicine (Table 2). Most of the wound-healing information was collected from recent literature from the last 10 years. The Euphorbiaceae family was the least represented (five members), followed by the Asteraceae family (six members) and the Fabaceae family (eight members). The most commonly used plant parts were cited as leaves (37%), followed by fruits (9%), seeds (8%), roots (8%), aerial parts (7%), flowers (6%), the whole plant (6%) bark (5%), saponins (3%), rhizome (2%) and others. These data also show that medicinal plants are used to treat wounds in many different parts of the world. Different families and genera have been analyzed in this work as regards their components. In 36% of the genera, the major phytochemical compounds found were alkaloids, steroids, flavonoids, saponins, tannins, and terpenes.

## 5. Bioactive Phytocompounds with Wound-Healing Properties

**Figure 2:**
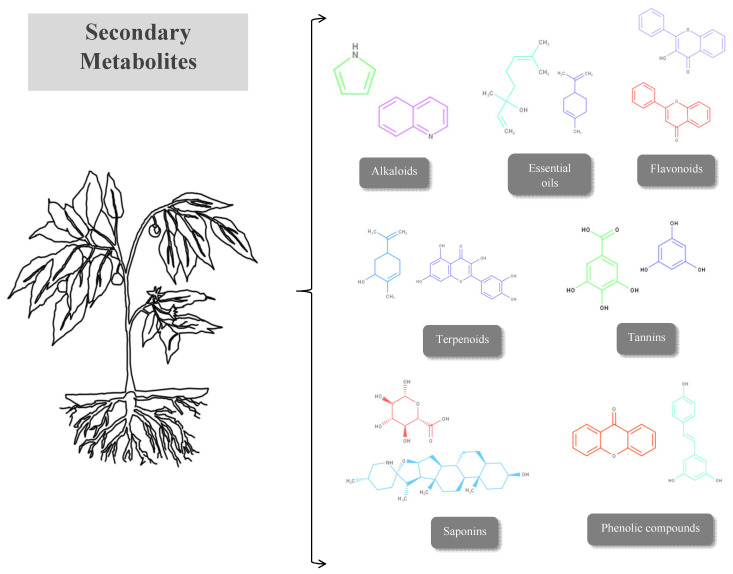
Secondary metabolites.

In the relevant literature, a variety of studies have addressed different plants with wound-healing properties. These studies have described the pharmacological activities of plants employed in wound healing and their molecular mechanisms to validate their traditional use and development into safe and effective herbal treatments for wounds. Due to the plants’ metabolism, secondary metabolites can be considered as bioactive molecules with therapeutic potential of great value in the pharmaceutical, cosmetic, and food industries, as concerns the design and formulation of medicines for different illnesses with less severe side effects [148,149,150,151,152]. The bioactive phytochemical compounds found include secondary metabolites such as alkaloids, essential oils, flavonoids, tannins, terpenoids, saponins, and phenolic compounds [153,154,155,156] (Figure 2). 

**Figure 3:**
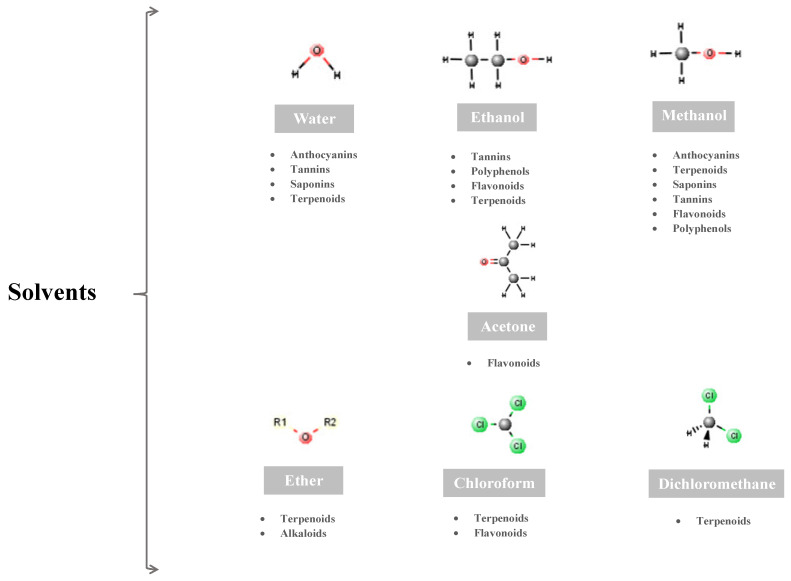
Selective solvents and groups of metabolites.

The allocation of these active compounds into different plant parts, as has been widely described, involves the use of different selective solvents to derive complex mixtures of groups of metabolites (Figure 3).

**Figure 4:**
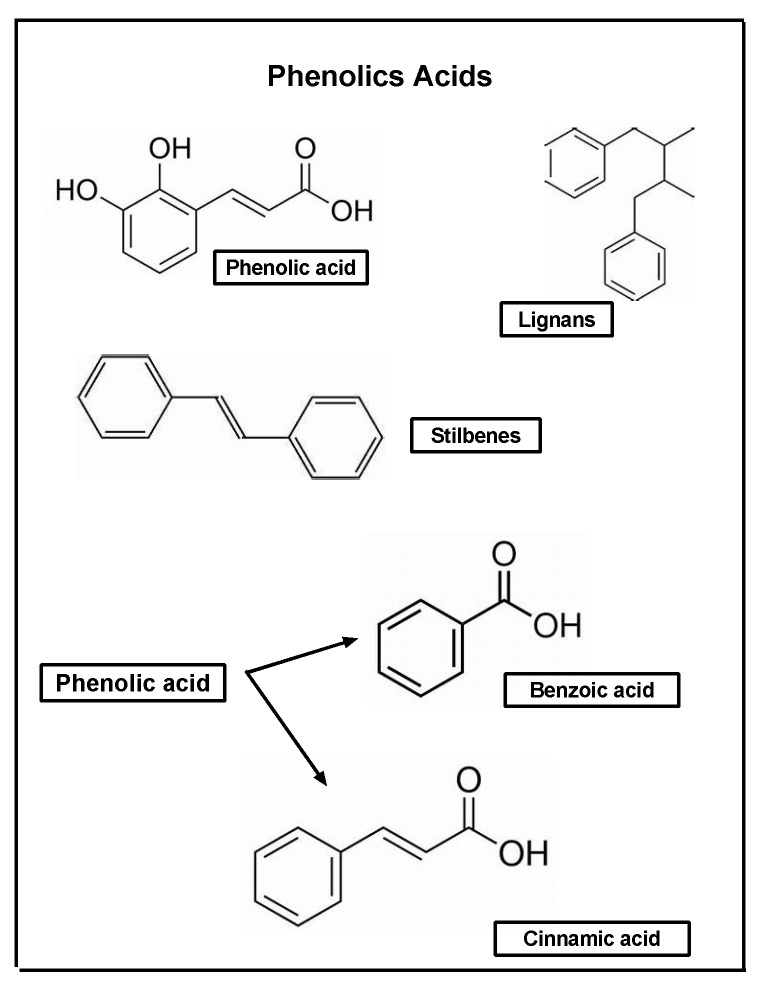
General structure of phenolic acids.

Phenolic acids are the bioactive compounds most widely found in legumes, cereals, vegetables, and fruits. They are also responsible for certain characteristics of foods, such as aroma and astringency, as well as color and flavor [153,155,156,157] (Figure 4). 

**Figure 5:**
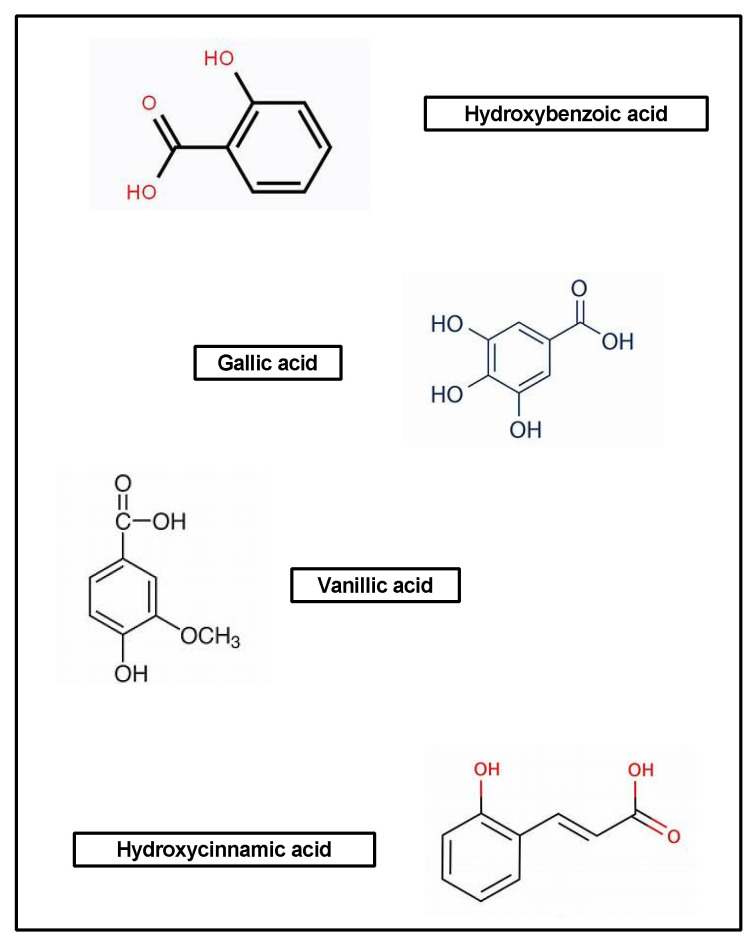
Other phenolic compounds.

These compounds also play a role in plants’ ability to protect themselves against different insults, such as ultraviolet radiation and pathogens [153,156,157]. Importantly, the amounts of phenolic compounds produced by plants can vary according to environmental conditions, genetic factors, and degree of maturation [158]. In the literature, it has been described how phenolic compounds act as anti-inflammatory and antiproliferative agents, antioxidants, transduction modulators, stimulants of collagen production, and antimicrobials, in addition to carrying out other functions [154,156,157]. These compounds can be categorized into hydroxybenzoic acids, such as gallic and vanillic acids, as well as hydroxycinnamic acids such as ferulic and caffeic acids. They have also been shown to have immunomodulatory, antioxidant, hepatoprotective, and anti-inflammatory actions [159] (Figure 5).

Potent antioxidant agents, such as flavonoids, act as reducing agents and protect against radiation [160,161]. These protective effects mean that they can modulate pro-inflammatory molecules, such as those involved in the healing process [160,162]. The effects of flavonoids in the inflammatory process extend to the treatment of diseases linked to inflammation and processes of which inflammation is a part, such as the healing process and the inhibition of invasion, angiogenesis, and metastasis mechanisms [160]. 

**Figure 6:**
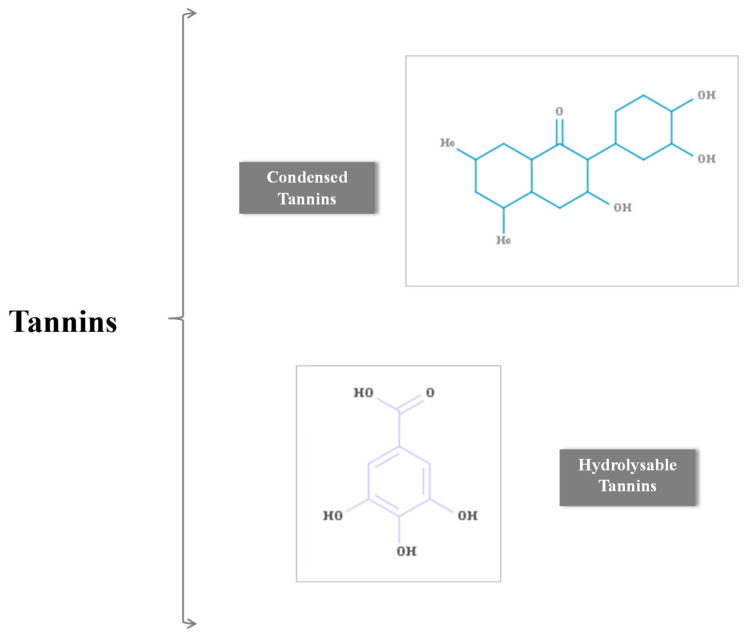
Hydrolysable and condensed tannins.

Tannins are the most complex of the phenolic compounds, categorizable as condensates or hydrolysable. Their role is to protect plants from pathogens through protein complexation, and via their antimutagenic activity, they promote healing through the modulation of different cellular mechanisms and growth factors [153,156,157] (Figure 6).

## 6. Activity of Bioactive Phytochemicals in Wound Healing

Impaired vascular function, ischemia, superficial debris, and necrosis are the main factors that cause poor immune responses and, consequently, contribute to the development of continued chronic wounds. Excessive bacterial growth and the formation of a biofilm lead to a chronic and self-perpetuating inflammatory state via the modification of aspects of the wound microenvironment, such as its humidity, pH, metalloproteinases, and reactive oxygen species. As many of these microenvironment-related factors as possible must be taken into account to develop beneficial therapeutic strategies [163]. Nature, as described in the literature, is a rich source of therapeutic possibilities. Secondary metabolites can promote the wound-healing process through their pharmacological effects on the body. These compounds include phenolics, alkaloids, and fatty acids, as well as glycosylates and polysaccharides. Such compounds have also been confirmed to have beneficial effects related to their anti-inflammatory, antioxidant, and antibacterial properties, and they promote collagen synthesis and facilitate protective cell regeneration [164,165,166]. In addition, these active compounds present low toxicity and good absorption by the skin barrier [164]. The improved efficiency of treatments using natural extracts is related to the establishment of synergy, which enhances the effects of products of natural origin as well as current therapeutic approaches. Various studies have demonstrated that such synergistic interaction is a result of these substances’ antibacterial, antioxidant, and anti-inflammatory properties [167]. Active research in this area is currently focused on developing wound treatments able to prevent microorganisms from entering wounds with a bactericidal effect. Recent studies have shown that the use of vegetal extracts and their secondary metabolites has been integrated into diverse treatment modalities, and this has been proven to be effective against both Gram-positive and Gram-negative bacteria [168]. Some have already been selected for use in clinical trials or incorporated into nanoparticles [169]. Studies have shown that natural metabolites can represent beneficial candidates for use in wound healing. One obstacle in developing their clinical use is their poor oral or topical bioavailability.

### 6.1. Essential Oils 

Research has shown that volatile essential oils present a variety of beneficial properties, such as antioxidant, antiviral, anticancer, insecticidal, anti-inflammatory, antiallergic, and antimicrobial effects [168]. These mixtures of lipophilic components are considered safe and biocompatible, although due to their low water solubility, bioavailability, and stability, their therapeutic uses can be limited [169]. 

### 6.2. Polyphenols 

Polyphenols are considered multifaceted agents due to their beneficial activities, such as antibacterial, anticancer, anti-inflammatory, and antioxidant effects, in addition to their complex wound-healing properties [170]. However, the main problems include their hydrophobicity and poor water solubility, permeability, and bioavailability. 

### 6.3. Flavonoids

As an exemplary flavonoid, quercetin has been harnessed for its antibacterial, anti-inflammatory, and antioxidant activities. When converted into quercetin nanofibers, it provides a large porous surface area that can carry many active compounds that facilitate penetration into the skin. Trials conducted with quercetin patches have shown them to have antibacterial activity that combats acne [171]. In other trials, film structures of N-carboxybutyl chitosan (CBC) and agarose were analyzed for their potential utilization in topical membranous wound treatment. Other research has demonstrated the use of polymeric biomaterials loaded with quercetin and thymol. These have been utilized both individually and in the form of mixtures of these two substances, which have anti-inflammatory and anesthetic properties. The incorporation of quercetin into semisolid bases such as creams and acid carbomer gels has been proposed to investigate the effects of additives such as propylene glycol and polyethylene glycol on its release and skin retention. With respect to quercetin and chrysin, or quercetin within chitosan nanoparticles, propylene glycol is an absorption accelerator that can also prolong the antioxidant activity [172,173]. Another study has demonstrated that polymeric nanoparticles can enhance antiradical activity, along with chelating quercetin and catechin [174]. Other studies have demonstrated the additional benefits offered by apigenin to the skin via the stimulation of epidermal differentiation, the synthesis and secretion of lipids, and cutaneous antimicrobial production. In vitro studies have demonstrated that hesperidin and naringin obtained from citrus fruits can be used to synthesize stabilized nanoparticles in a green manner [174]. 

Phytochemicals have been described to enhance the effects of antibiotics due to their low toxicity and anti-infective, anti-inflammatory, and antioxidant properties [175]. They can act as efflux pump inhibitors, preventing biofilm formation or targeting specific bacterial virulence factors [175]. Research confirms that plants from different families can facilitate the healing process and attenuate inflammation [176,177,178]. Certain compounds, including the flavonoid baicalein and the monoterpene phenol thymol, have an inhibitory effect on inflammation that has been demonstrated in mixtures of ethanol and can act synergistically, suggesting their use as an alternative treatment to antibiotics [176,177,178]. 

## 7. Mechanisms of Effects of Phytochemicals on Wound-Healing Agents

### 7.1. Antioxidant Activities of Wound-Healing Agents

Large amounts of energy must be produced for normal cellular activities, which is achieved through mechanisms such as oxidation, resulting in the generation of reactive oxygen species (ROS) and reactive nitrogen species (RNS) [156]. These reactive species possess unpaired electrons in their valence shell and are unstable [154]. Radical species include hydroxyl radicals (OH^−^), nitric oxide radicals (NO), singlet oxygen (^1^O_2_), and superoxide radicals (O_2_^−^). These are produced naturally in the body, but adverse factors such as stress and pollution can increase their levels, causing them to damage molecules such as proteins and DNA, leading to the disintegration of cell membranes. Oxidative stress is strongly linked to the development of chronic diseases and aging [154,157,160]. 

The use of antioxidants to control the levels of reactive species in the body is recommended. Antioxidants are defined as substances with the capacity to control the oxidation of biomolecules and act in the sequestration of reactive species such as ROS or RNS; some can also chelate metal ions and modulate enzymes related to oxidative stress [154,156]. Such enzymes include catalase (CAT), which catalyzes the degradation of hydrogen peroxide (H_2_O_2_), and glutathione peroxidase (GPx), which removes hydroperoxides [154].

Non-enzymatic processes involve transferrin, reduced glutathione (GSH), ubiquinol, and melatonin [154,156]. The antioxidant effects of these compounds are related to the presence of phenolic compounds, amino acids, sterols, ascorbic acid, peptides, and phospholipids in their composition [179]. Several studies have shown that antioxidants have anti-inflammatory, vasodilatory, antitumor, antiallergic, antiviral, and cardioprotective activities, among other properties [156,157,179]. 

During the healing process, excess free radicals are produced at the site of injury. This can be limited by the presence of antioxidants, which prevent some of the damage caused to cells [180]. The antioxidant action of medicinal plants is strongly related to the quantities of bioactive compounds they contain, such as flavonoids, which act as antioxidants and also directly participate in the inflammatory phase, limiting cellular damage due to their effects on prostaglandins and macrophages [181]. Flavonoids are also capable of increasing the resistance of collagen fibers, thus facilitating the process of the contraction and re-epithelization of wounds [180,182]. 

The healing process restores tissue integrity when an injury occurs [183]. It can be impeded by factors such as diabetes, which causes it to be slower and less efficient, thus potentially causing chronicity [184,185]. Poor healing can lead to the loss of tissue function, the chronification of injuries, and amputation, and it can also produce physical, psychological, social, and economic damage [184]. The treatments used to promote healing include the use of natural products and their derivatives. Some of the medications currently available are not completely effective in treating chronic wounds. For this reason, it is essential to continue research into new substances with more effective healing properties. Flavonoids and tannins have shown antiproliferative properties and are capable of regulating the production of free radicals; they are also involved in limiting inflammatory mechanisms [180]. Further investigations in the pharmaceutical, food, and cosmetic sectors will be essential in addressing the sources of antioxidants and substances that can be used to treat certain conditions, such as chronic wounds and cancer. Following the formation of ROS, the wound-healing process is significantly delayed; however, their formation is limited by the presence of flavonoids, which are responsible for increasing the levels of common antioxidant enzymes. The use of flavonoids in the clinical setting is very limited due to their low bioavailability. An important property of flavonoids obtained from plants is their lipophilicity against Gram-positive bacteria, which is a product of their involvement in the damage done to the respiratory chain and other aspects [186]. 

### 7.2. Anti-Inflammatory Properties of Wound-Healing Agents

**Figure 7:**
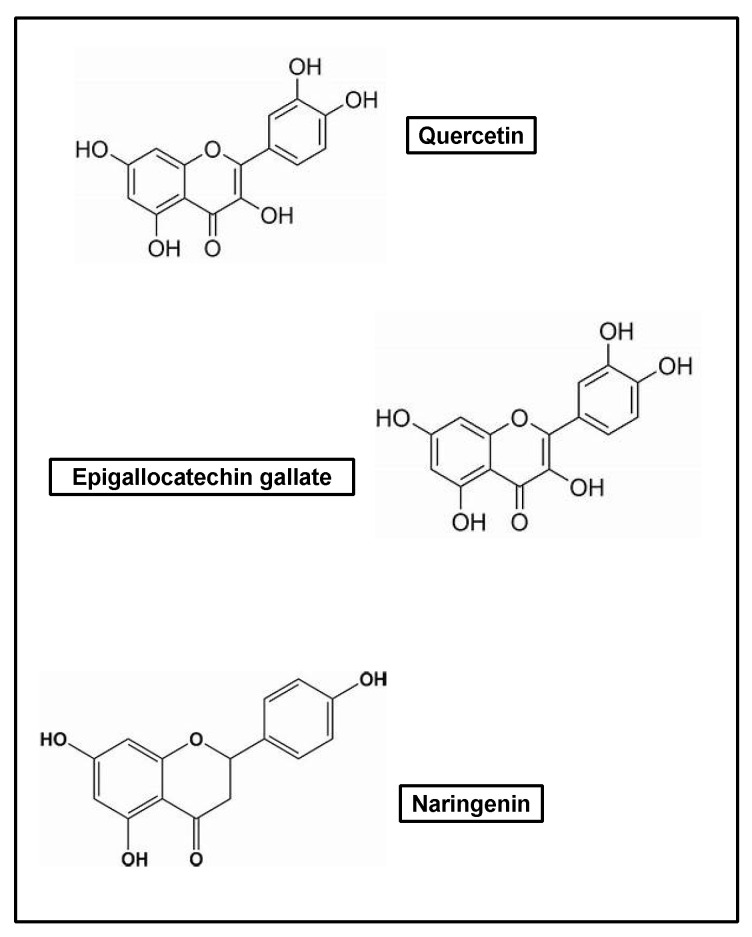
Most widely studied flavonoids.

Flavonoids have also been suggested as a candidate for use in the treatment of a variety of skin lesions, with minimal side effects when administered by topical application due to their lipophilic nature [187]. The many properties exhibited by flavonoids, such as their anti-inflammatory, antimicrobial, and antifibrotic effects, can be understood as a result of their polyhydroxy structure. Among all the structurally different flavonoids, twenty-four have demonstrated the ability to accelerate the healing process, and the most studied are quercetin, epigallocatechin gallate, and naringenin [55] (Figure 7).

Numerous studies have also shown that flavonoids are capable of decreasing the levels of inflammatory mediators, such as prostaglandins and leukotriene, and pro-inflammatory cytokines, such as IL-1β, TNF-α, IL-6, and IFN-γ. They can also increase the production of anti-inflammatory mediators, such as interleukin 10 (IL-10), negatively regulate the expression of nuclear factor kappa B (NF-κB), and block cyclooxygenase activity.

**Figure 8:**
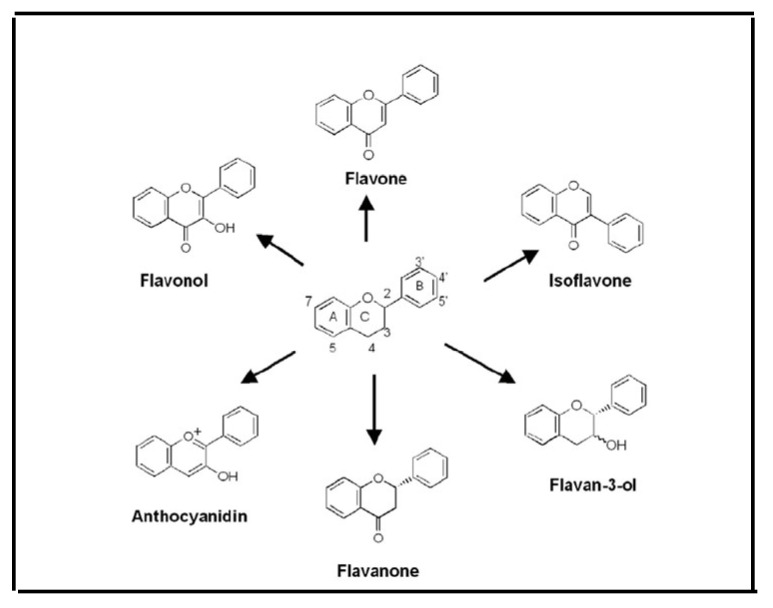
Prenylated flavonoids.

Prenylated flavonoids are found in plants’ roots, bark, seeds, and buds. These are part of a subclass of modified flavonoids with at least one lipophilic side chain of variable length, and they possess favorable biological activities, such as antimicrobial, antifungal, larvicidal, estrogenic, osteogenic, immunosuppressive, anticancer, anti-inflammatory, antioxidant, antiallergic and cytotoxic effects [188]. The group of prenylated flavonoids includes C-prenylated chalcones/dihydrochalcones, flavanones, flavones, flavonols, isoflavones, and, less frequently, O-prenylated forms (Figure 8).

These structures can be replaced, following oxidation, reduction, dehydration, and/or cycling, with 3,3-dimethylallyl, 1,1-dimethylallyl, geranyl, lavandulyl, and farnesyl side chains [188]. Studies have shown the advantages offered by prenyl compared to flavonoids. Prenylated flavonoids have a greater affinity with the cellular membrane and P-glycoprotein inhibitors [189] and show antibacterial and inhibitory or enzyme-enhancing actions, while prenylation causes an increase in lipophilicity and the affinity for biological membranes [188,189]. 

Diplacone, with its 6-geranyl-30,40,5,7-tetrahydroxyflavanone structure, has shown anti-inflammatory properties both in vitro and in vivo, with different mechanisms of action. It can cause reductions in TNF-α and MCP-1 expression and regulates the expression of zinc-finger protein 36, which increases cytokine degradation [190]. Another compound, isobavachalcone, suppresses the production of nitric oxide and negatively regulates inflammation-related enzymes such as iNOS and 15-LOX [191,192]. Licochalcone A is a 5-(2-methylbut-3-en-2-yl) chalcone obtained from licorice roots, and it has been traditionally used to treat inflammatory diseases. It inhibits the activation of transcription factors such as NF-κB and AP-1; it also suppresses pro-inflammatory cytokines and NO and PGE2 production [193,194]. The main function of Sophoraflavanone G is the inhibition of eicosanoid-forming enzymes [195]. It can also disrupt NF-κB and MAPK signaling pathways [195,196]. Another prenylated chalcone is xanthohumol, which is found in *Humulus lupulus* L. hops and has antistaphylococcal activity [197,198]. Its anti-inflammatory effect is enacted through the inhibition of NO levels due to the suppression of inducible NO synthase, and it inhibits both the activation of NF-κB [199,200] and the production of the cytokines MCP-1, TNF-α, and IL-12, as well as oxidative stress [201,202,203]. 

### 7.3. Antimicrobial Effects of Wound-Healing Agents

In the previous sections, we mention that flavonoids are widely used as effective therapeutic agents and that numerous in vitro and in vivo studies have confirmed them to have important functions, mainly defensive and regulatory [203]. Regarding their functions as protective agents against microorganisms, flavonoids act directly on bacterial cells, as well as suppressing virulence and the formation of biofilms. They can also act synergistically with antibiotics [204]. These properties have enabled the production and use of semisynthetic or synthetic flavonoids to combat microorganisms [205]. 

The antibacterial activities of flavonoids and prenylated flavonoids are due to the structure of 2-phenyl-1,4-benzopyrone, which has been suggested to be capable of influencing different cellular processes [204]. Apigenin and quercetin present the ability to inhibit bacterial cell walls by inhibiting D-alanine–D-alanine (D-Ala–D-Ala) ligase, which is crucial to the completion of peptidoglycan precursors [206]. Researchers have indicated that several flavonoids can modify membrane permeability and damage membrane functions. On the other hand, flavanols, flavolans, and green tea catechins have been shown to disturb bacterial cytoplasmic membranes through hydrogen peroxide [207,208]. Another flavone, Artocarpin, obtained from the Moraceae family, with prenyl in position 3 and a (1E)-3-methylbut-1-enyl moiety in position 6, presented remarkable antibacterial activity [197,209,210,211].

## 8. Conclusions

Plants are excellent wound healers, and when used in the context of different wound models, they can be employed as part of proper measures to treat wounds and control the healing process. Thus, herbal medicines have gained popularity in several countries. The factors that must be considered in the healing of a lesion are the wound closure rate, epithelialization, tensile strength, histopathology, and granuloma weight. This study discusses how traditional medicines could play important roles in wound healing. Modern knowledge of these bioactive principles can provide alternatives to improve or accelerate wound healing with minimal toxicity. The preliminary evidence and results in the current literature suggest that this is an active area of study. In future studies, factors such as the potential toxicity to human cells, kinetics and speed of healing, wound types, chronicity, timing of application, and dose of therapeutic agent must be considered. The preparation of formulations that include medicinal plants as part of their release and distribution systems for their anti-inflammatory, antioxidant, and wound-healing properties requires further investigation. These proposed studies on natural or synthetic formulations can be achieved by acquiring certain quantities of pure compounds and their extracts for standardization.

## Figures and Tables

**Table 1:** Classification of a wound.

Classification	Type
Cause	Pathological: resulting from a pathology (pressure ulcer, neoplasia). Surgical or traumatic: resulting from surgery or trauma. Iatrogenic: resulting from procedures or treatment with radiotherapy.
Evolution	Acute: wounds of easy resolution, rupture of vascularization, and mediate triggering of homeostasis (cuts, scoring, burns). Chronic: long-lasting wounds (deviation from the physiological cicatricial process).
Presence of infection	Clean: free of microorganisms. Clean-contaminated: lesions less than 6 h between trauma and initial care. Infected: presence of local infectious agent. Contaminated: wounds serviced more than 6 h after trauma.
Regarding tissue impairment	Stage I: skin integrates with signs of hyperemia, discoloration, or hardening. Stage II: the epidermis and dermis are ruptured, with subcutaneous tissue showing hyperemia, blisters, and a shallow crater. Stage III: total loss of cutaneous tissue, necrosis of the subcutaneous tissue to the muscular fascia. Stage IV: great tissue destruction with necrosis reaching muscles, tendons, and bones.
Degree of openness	Open: wounds in which the edges of the skin do not touch. Closed: wounds where the edges of the skin are juxtaposed.

**Table 2:** Families and Genus with wound healing activity.

Family	Genus	Part Used/Type Extraction	Compounds	Ref.
*Acanthaceae*	*Justicia flava*	Leaf/Methanol	Alkaloids, Flavonoids, Glycosides, Tannins	[41]
	*A. paniculata*	Leaf/10% aqueous extract	Diterpenoids	[42]
* Amaranthaceae *	* Achyranthes aspera *	Leaf/Ethanol	Flavonoids, Saponins, Tepernoids	[43]
	* A. sessilis *	Stem and Leaf/Methanol	2,4-dihydroxy-2,5-dimethyl-3(2H)-furan-3-one, hexadecanoic acid, 2-1,2,4-trioxolane,3-phenyl-, palmitate-ethyl-, L-glutamic acid.	[44]
	* A.triandra *	Air seed/Petroleum ether	Oil ricinoleic acid	[45]
	* Celosia argentea *	Root/Dichloromethane and ethyl acetate	Terpenoids	[46]
* Anacardiaceae *	* Buchanania lanzan * S.	Root/Petroleum ether	Alkaloids, Flavonoids, Polyphenols, Steroids	[47]
	* Lannea welwitschii * Hiern	Leaf/Methanol	Alkaloids, Flavonoids, Glycosides, Steroids, Tannins	[41]
* Apiaceae *	* Angelica sinensis *	Leaf/Ethanol	n-buthylidenephthalide and proteins	[48]
	* Centella asiatica *	Leaf/Methanol	Asiaticoside, Madecassic acid, Madecassoside asiatic acid, Triterpenes,	[49]
	* Cuminum cyminum *	Seeds	Essential oils	[50]
	* L. striatum *	Rhizoma	Essential oils	[51]
* Apocyanaceae *	* Catharanthus roseus *	Leaf/Aqueous and Methanol	Alkaloids, Phenols, Proteins, Saponins, Tannins	[52]
	* S. hispidus *	Leaf and Root	Alkaloids, Flavonoids, Saponins, Tannins,	[53]
	* Wrightia tinctoria *	Leaf/Aqueous	Alkaloids, Flavonoids, Phenolics, Saponins, Tannins	[54]
	* Saba florida *	Leaf/Methanol 99.9%	Total extract	[55]
*Araliaceae*	*Panax ginseng*	Panax ginseng saponins (PGS)	Ginsenoside Rb1 (G-Rb1)	[56]
	*Panax notoginseng*	Panax notoginseng saponins (PNS)	High-glucose (HG-30Mn)	[57]
*Asclepiadaceae*	*Calotropis giganthea*	Root Bark	Taraxasteroryl isovalerate, Gigantin, Giganteol, Isogiganteol, α-amyrin-3-amyrin, Taraxasterol	[58]
	*Calotropis procera*	Root bark/Ethanol	Alkaloids, Flavonoids, Steroids, Tannins	[59]
*Asphodelaceae*	*Aloe vera*	Leaf/Acetonic extract	Polymers	[60]
* Asteraceae *	* Achillea millefolium *	Aerial parts	Yarrow Oil	[61]
	* Arctium lappa *	Ground bark/Ethanol	Alkaloids, Flavonoids, Lignans, Phenolic acid, Tannins, Terpenoids	[62]
	* Blumea balsamifera *	Leaf/Methanol 95%	Flavonoids, Nonvalatile constituents	[63]
	* Calendula officinalis *	Flowers/Hydroethanol	Rutin, Quercetin-3-O-glucoside	[64,65]
	* Carthamus tinctorius *	Saflowers	Hydroxysaflow yellow A (HSYA)	[66]
	* Wedelia trilobata *	Leaves/Ethylacetate, Chloroform:Methanol	Kaura-9(11),16-dien-19-oic acid	[67]
* Bignoniaceae *	* Kigelia africana *	Leaves/Roots/Methanol	Flavonoids, Carbohydrates, Sapogenetic glycosides, Saponins, Steroids	[68]
	* S. campanulata *	Leaf/Methnol	Flavonoids, Phenols, Saponins, Steroids	[69]
	* Tecoma capensis *	Shoots/Hydroalcoholic	Myrecetin	[70]
* Boraginaceae *	* H. indicum *	Leaf/Ethanol	Crude extract	[71]
	* L. erythrorhizon *	Root	Purification of Shikonin	[72]
* Burseracea *	* Boswelia sacra *	Leaf/Methanol	Oil	[73]
	* C. myrrha *	Leaf/Methanol	Oleo-gum-resins	[73,74]
*Cactaceae*	*O. ficus-indica*	Seed/Oil extraction	OFI-SNEDDSs	[75]
*Caricaceae*	*Carica papaya*	Papaya fruit extraction	Crude extract	[76]
* Cecropiaceae *	* Cecropia peltata *	Leaf	Saponins	[77]
	* Myrianthus arboreus *	Leaves/Ethanol	Alkaloids, Flavonoids, Glycosides, Sterols, Tannins, Terpenoids	[78]
*Caprifoliacea*	*Locinera japonica*	Flowers/Ethanol	Chlorogenic acid	[79]
* Combretaceae *	* C. mucronatum *	Leaf/Ethanol	Procyanidin B2	[80]
	* Terminalia chebula *	Fruit extraction	Anthraquinone, Flavonoids, Sapogenins, Saponins, Steroids, Tannins	[81]
	* Terminalia arjuna *	Fruit extraction/Methanol	Anthraquinones, Carbohydrates, Flavonol, Glucose sorbitol, Hydrolyzable Tannins	[82]
* Crassulaceae *	* Bryophylum pinnatum * Lam	Leaf/Aqueous	Patulitin-O-deoxy-hexoside-O-hexoside, Quercetin-O-hexoside, Quercetin-O-deoxy-hexoside-O-pentoside	[83]
*Cyperacea*	*Cyperus rotundus L*	Aerial part/Methanol	Alkaloids, Phenols	[84]
* Euphorbiacea *	* Alchornea cordifolia * (Schum & Thonn)	Leaf/Ethanol	Quercetin, Hyperin, Guaijaverin	[78]
	* Euphoria hirta *	Whole plant/Methanol	Alkaloids, Flavonoids, Glycosides, Proteins, Saponins, Tannins	[85]
	* Jatropha curcas * L.	Flowers/Methanol	Alkaloids, Flavonoids, Glycoside, Saponins, Tannins	[86]
	* Mallotus oppositifolius * (Geiseler)	Leaf/Ethanol	Aspinidiol B, methylene bis-aspidinol, α-tocoferol	[87]
	* P. emblica * L.	Leaves/Ethanol	Flavonoids, Saponins, Tannins	[88]
	* P. muellerianus * (Kuntze)	Leaf/Aqueous	Geranin	[89]
* Fabaceae *	* Astragalus membranaceus * Sprants	Seeds/Ethanol	Tryptophan, Linoleic acid, Adenine	[90]
	* Caesalpinia sappan * L.	Wood	Sappachalcone	[91]
	* Entada phaseoloides *		Total Tannins	[92]
	* Glycyrrhiza glabra * L.	Root/Ethanol	Glycyrrhiza cream	[93]
	* Indigofera enneaphylla * L.	Whole plant/Petroleum ether, Ethyl Acetate, Ethanol	Flavonoids, Saponins, Tannins	[94]
	* Mimosa pudica * L.	Seeds/Ethyl Acetate Root/Petroleum ether	Alkaloids, Glycosides, Phytosterol	[95,96]
	* Sophora flavescens *		Compound, Sophora flavescen lotion	[97]
	* Tephrosia purpurea *	Aerial plants/Ethanol	Flavonoids, TPF-A 7 peaks	[98]
*Fagaceae*	*Quercus infectoria* Oliver	Nutgails/Ethanol	Pharmaceutical formulations	[99]
*Ganodermataceae*	*Ganoderma lucidum*	Fruting bodies/Hot water	Polysaccharides 25.1% Ganodermic acid A	[100]
* Gentianaceae *	*Anthocleista nobilis* G. Don	Stem bark/Ethanol Ethyl Acetate Buthanol n-Hexane	Isovitexin and Isovitexin-2”-O-xyl Isovitexin Apigenin monoglycoside p-Hydroxybenzoic acid, Sarasinside	[101]
* Ginkgoaceae *	* Ginkgo biloba * L.	Leaf/Aqueous	Myricerin, Quercetin, Kaempferol, Isorhamnitin, Terpenes lactones, Ginkgolic acid	[102]
* Hypericaceae *	*Hypericu mysorense*	Parts plant/Methanol	Flavonoids, Saponins, Tannins	[103]
*Iridaceae*	*Crocus sativus* L.	Stigmas/Glycerin/water/Ethanol	Flavonoids, Anthocyanins	[104]
*Lamiaceae*	* Occimum sanctum * L.	Leaf/Water	Essential Oil	[105]
	* Rosmarinus officinalis *	Aerial parts/Hydrodistillation	Essential Oil	[106]
	* Salvia miltiorrhiza *	Leaf/Hydroethanolic	Flavonoids, Total Phenols	[107]
* Lauraceae *	* Cinnamomum cassia *		Cinnamon Oils	[74]
* Liliaceae *	* Allium cepa * L.	Onion/Ethanol 95%	Alkaloids, Flavonoids, Phenols, Tannins	[108]
* Lycopodiaceae *	* Lycopodium serratum *	Aerial parts/Ethanol	Crude etanol extract	[109]
* Lythraceae *	* Lawsonia alba *	Leaf/Methanol	Coumarin, Flavonoid, Steroid, Tannin, Terpenoid	[110]
	* Lawsonia inermis * L.	Leaf/Aqueous	Total Phenols, Total Flavonoids, Total Tannins, Saponins	[111]
	* Punica granatum * L.	Fruit whole	Pomegranate are Tannins, Flavonoids, Punicic acid, Phytoestrogen	[112]
* Malvaceae *	* Hibiscus rosa sinensis * L.	Flowers/Methanol	Phenolic compounds, Flavonoids, Essential Oils, Anthocyanins	[113]
	* Malva sylvestris *	Flowers/Ethanol:Water (80:20)	Total phenolic, Flavonoids, Anthocyanin	[114]
	* Thespesia populnea * L.	Fruit/Aqueous	Glycosides, Flavonoids, Alkaloids, Phytosterol, Quercetin, Rutin, Lupeal	[115]
* Martyniaccae *	* Martynia annua *	Leaf/Ethanol	Glycosides, Phenols, Flavonoids, Tannins, Anthocyanins MAF-C 7 peaks	[98]
* Meliaceae *	* A. indica * A. Juss	Steam bark/Water:Ethanol	Crude	[116]
	* Carapa guianensis * Aubl	Andiroba seed oil	Lauric axid, Myristic, Palmitic acid, Stearic acid, Oleic acid, Linoleica cid, Lignoceric acid, Palmitoleic acid, Heptadecanoic acid, Arachidic acid, Behenic acid	[117]
* Mimosaceae *	* Prosopis cineraria *	Leaves/Petroleum ether	Protocatechuic acid, Caffeic acid, Chlorogenic acid, Ferrulic acid	[118]
* Moraceae *	* Ficus religiosa * L.	Leaves/Methanol	Glycosides, Alkaloids, Tannins, Terpenoids	[119]
* Moringaceae *	* Moringa oleífera * Lam.	Leaves/Ethanol	Flavonoids, Phenolic acids	[120]
* Musaceae *	* Musa sapientum * L.	Fruits/Ethanol	Saponins, Flavonoids, Glycosides, Steroids, Alkaloids	[121]
* Myrsinaceae *	* Embelia ribes * Burn.	Fruits/Petroleum ether	Embelin	[122]
* Myrtaceae *	* Eucalyptus globulus *	Leaves/Hydrodistillation	1,8-cineole content 72.3%, α-pinone 9.4%	[123]
* Nymphaeaceae *	* Nelumbo nucifera *	Aerial part/Ethanol	30 peaks Ethanol,2-(-Octadecinyloxy, γ-sitosterol, Hexadecanoic acid	[124]
* Oleaceae *	*Jasminum auriculatum* Vahl.	Leaves/Petroleum ether	Alkaloids, Carbohydrates, Flavonoids, Phenolic compounds, Saponins, Steroids, Tannins, Tepernoids	[125]
	*Jasminum grandiflorum* L.	Leaves/Methanol	Crude	[126]
* Orchidaceae *	* Bletilla striata *	Root/Boiled water	Polysaccharide content (65.3%)	[127]
* Paeoniaceae *	* Paeonia suffruticosa *	Bark root/Alcohol	Flavonoids, Phenolic acid, Polysaccharide, Saponins	[128]
* Papaveraceae *	* Argemone mexicana * L.	Fruits/Methanol	Alkaloids, Flavonoids, Glycosides, Saponins, Steroids, Tannins, Terpenoids	[129]
* Papilionaceae *	* Trigonella foenum-graecum *	Aerial part/Methanol	Flavonoids	[130]
* Pedaliaceae *	* Sesamum indicum *	Seed/Ethanol	Sesame Oil	[131]
* Plantaginaceae *	* Plantago *	Leaves/Distilled water	Polyphenolic compounds	[132]
*Polygonaceae*	* Rheum officinale *	Powders/Ethanol	TMC extracts	[133]
* Potulacaceae *	* Portulaca grandiflora *	Total plant/Ethanol	Alkaloids, Flavonoids, Saponins, Terpenoids	[134]
* Phyllanthaceae *	* Bridelia ferruginea * Benth.	Leaves/Methanol Stem barks/Ethyl Acetate	High phenolic content High flavonoids content	[135]
* Rosaceae *	* Sanguisorba officinalis *		Polysaccharide	[136]
* Rubiaceae *	* Morinda citrifolia * L.	Leaf	Alkaloids, Coumarins, Flavonoids, Saponins, Tannins, Triterpenes	[137]
	* Rubia cordifolia * L.		100 Compounds bicyclic peptides, terpenes, polysaccharides, Flavonoids, Quinones	[138]
* Rutaceae *	* Aegle marmelos * L.	Flower/Ethanol 60%	Aegelin, Cineol, Cuminaldheyde, Luvangetin, Eugenol	[139]
	* Zanthoxylum bungeanum * Maxim	140 constituents of this plant	Alkaloids, Fatty acids, Flavonoids, Tepernoids, Flavonoids	[140]
* Salicaceae *	* Casearia sylvestris *	Leaves/Hydroalcoholic	Crude extract	[141]
* Scrophulariaceae *	* Rehmannia glutinosa *		Polysaccharides	[142]
* Stemonaceae *	* Stemona tuberosa *		9,10-dihydro-5-methoxy-8-methyl-2,7-phenanthrenediol	[143]
* Theaceae *	* Camellia sinensis *	Tea leaves/Methanol		[144]
* Thymelaeaceae *	* Daphne genkwa * Sie.		Diterpenoids/yuanhuapine	[145]
* Vitaceae *	* Ampelopsis japonica *	Root/Methanol	Catechin, Gallic acid, Kaempferol, Euscaphic acid, Resveratrol, Epicatechin	[146]
* Zingiberaceae *	* Curcuma longa * Linn	Extracts	Alkaloids, Flavonoids, Phenolic, Saponins, Terpenoids, Steroids	[147]

## Data Availability

Data sharing is not applicable.
